# A novel male Japanese quail structural connectivity atlas using ultra-high field diffusion MRI at 11.7 T

**DOI:** 10.1007/s00429-022-02457-2

**Published:** 2022-03-31

**Authors:** Raïssa Yebga Hot, Marine Siwiaszczyk, Scott A. Love, Frédéric Andersson, Ludovic Calandreau, Fabrice Poupon, Justine Beaujoin, Bastien Herlin, Fawzi Boumezbeur, Baptiste Mulot, Elodie Chaillou, Ivy Uszynski, Cyril Poupon

**Affiliations:** 1 Unité BAOBAB, NeuroSpin, Université Paris-Saclay, CNRS, CEA, 91191 Gif-sur-Yvette, France; 2 Unité de Physiologie de la Reproduction et des Comportements (PRC), INRAE, CNRS, IFCE, Université de Tours, 37380 Nouzilly, France; 3 UMR 1253, iBrain, Université de Tours, Inserm, 37044 Tours, France; 4 Zooparc de Beauval & Beauval Nature, 41110 Saint-Aignan, France

**Keywords:** Diffusion MRI, Japanese quail, Atlas, Ultra-high field, Ex vivo, Connectivity

## Abstract

The structural connectivity of animal brains can be revealed using post-mortem diffusion-weighted magnetic resonance imaging (MRI). Despite the existence of several structural atlases of avian brains, few of them address the bird’s structural connectivity. In this study, a novel atlas of the structural connectivity is proposed for the male Japanese quail (*Coturnix japonica*), aiming at investigating two lines divergent on their emotionality trait: the short tonic immobility (STI) and the long tonic immobility (LTI) lines. The STI line presents a low emotionality trait, while the LTI line expresses a high emotionality trait. 21 male Japanese quail brains from both lines were scanned post-mortem for this study, using a preclinical Bruker 11.7 T MRI scanner. Diffusion-weighted MRI was performed using a 3D segmented echo planar imaging (EPI) pulsed gradient spin-echo (PGSE) sequence with a 200 $\upmu$m isotropic resolution, 75 diffusion-encoding directions and a b-value fixed at 4500 s/mm^2^. Anatomical MRI was likewise performed using a 2D anatomical T_2_-weighted spin-echo (SE) sequence with a 150 $\upmu$m isotropic resolution. This very first anatomical connectivity atlas of the male Japanese quail reveals 34 labeled fiber tracts and the existence of structural differences between the connectivity patterns characterizing the two lines. Thus, the link between the male Japanese quail’s connectivity and its underlying anatomical structures has reached a better understanding.

## Introduction

The Japanese quail (*Coturnix japonica*) is a suitable farm animal to study cognitive functions and more particularly emotivity (Baer et al. 2015; Huss et al. 2008; Wilson et al. 1961; Lormant et al. 2018, 2020; Calandreau et al. 2011, 2013). Indeed, this bird presents an easily inducible and quantifiable response to fear called tonic immobility that has been described in the literature on animal behavior (Jones et al. 1994; Mills et al. 1997; Mills and Faure 1991). This innate behavior has been used to successfully produce two specific lines of Japanese quails: the short tonic immobility (STI) line and the long tonic immobility (LTI) line. One line has been selected for its long tonic immobility (TI) duration (the LTI line), the other one for its short TI duration (the STI line). It has been established that the STI and LTI lines, respectively, present low emotivity and high emotivity based on their behavioral and physiological responses (Bertin et al. 2018; Boulay et al. 2013; Faure et al. 2006; Jones et al. 1991; Richard 2000). Regarding structural connectivity, a few studies have been performed to map the structural connectivity in birds (De Groof et al. 2016; Güntürkün et al. 2013, 2020; Vellema et al. 2011; De Groof et al. 2006, 2016), and the Japanese quail might be an adequate species to finely map the structural connectivity in birds.

Diffusion MRI is a well-established technique to investigate the structural connectivity of in and ex vivo brains from animals to humans. Its principle is based on the observation of the microscopic movement of water molecules within the cerebral white matter consisting of bundles of axonal fibers organized in bundles. The membranes of the axonal fibers constitute obstacles that cause an anisotropy of water movement depending on the direction of space and thus indirectly reveal the direction of the bundles. The use of pulsed gradients tagging the position of the spins of the water molecules allows the MRI signal to be sensitive to this diffusion process (Stejskal and Tanner 1965; Le Bihan et al. 1986), and thus to access locally the direction of the axonal fibers. This local directional information can then be used to reconstruct virtual fibers representative of axonal pathways using so-called tractography algorithms (Basser et al. 1994; Poupon et al. 2000; Behrens et al. 2007; Parker et al. 2003; Mori and Zhang 2006; Parker et al. 2003; Reisert et al. 2011; Mangin et al. 2013) and, thus, infer models of the structural connectivity of any animal species.

While diffusion MRI is mostly used in vivo in humans, it is often used ex vivo in animals, because it allows the use of longer imaging protocols (from several hours to several days) to increase the quality of the acquired data. Indeed, ex vivo diffusion MRI is free of many sources of artifact generally corrupting the MRI signal in vivo, such as subject movement, physiological noise or susceptibility effects through the use of multi-shot echo planar sequences with a high number of shots allowing to decrease in the sensitivity to static field inhomogeneities and reach shorter echo times. In this way, it offers greater spatial resolutions, better signal-to-noise ratios (SNR), more diffusion directions, as well as more and higher achievable b-values than with living subjects. Nevertheless, ex vivo imaging protocols require monitoring the temperature during acquisitions, as well as the use of larger b-values to compensate for the strong reduction of the apparent diffusion coefficient (ADC) ex vivo (D'Arceuil and de Crespigny 2007) and thus preserve the diffusion contrast. The T_2_ relaxation time is also drastically reduced (Pfefferbaum et al. 2004; Shepherd et al. 2009) and narrows the window width available to acquire the data, reinforcing the need for stronger gradient magnitudes to keep echo times short and preserve the SNR.

dMRI on ex vivo animal and human brains has been the subject of many studies (D'Arceuil et al. 2007; Dyrby et al. 2011; McNab et al. 2009; Miller et al. 2011; Sébille et al. 2019), mostly focused on primates and rodents but few on birds. Previous studies in birds have used in vivo approaches to map the vocal pathways and functional vocal networks of song birds using manganese-enhanced MRI (Van Der Linden et al. 2004) and diffusion tensor imaging (DTI) (De Groof et al. 2006; De Groof and Linden 2010; Hamaide et al. 2020), but to our knowledge no ex vivo approaches involving both anatomical and diffusion MRI has been done to map the anatomy and structural connectivity of the male Japanese quail. However, a fine mapping of both in Japanese quails would allow to better capture the brain organization of this avian species.

This work is focused on the inference of the first structural connectome of the male Japanese quail. To this aim, ex vivo UHF MRI datasets were acquired on two cohorts of STI and LTI Japanese quails from which the first structural connectivity atlas of the Japanese quail was established. Then, to investigate the research hypothesis claiming that these two breeding lines do not show the same connectome, we studied the morphometric variability of all the white matter bundles available in the established connectivity atlas between the two lines.

## Materials and methods

### The Japanese quail cohort

21 male Japanese quails (*Coturnix japonica*) were scanned during the study and provided by the Pôle d’Expérimentation Avicole de Tours 103 (UEPEAT, INRAE 2018, Experimental Poultry Facility, 10.15454/1.5572326250887292E12), where they were bred and maintained. Male quails were selected since female individuals are subject to hormonal changes due to reproductive cycle and laying, which are likely to impact brain functions (Lormant et al. 2018). The Japanese quails that were involved in this study were bred in a closed building with a photoperiod 12 h/12 h. Consequently, they were not influenced by seasonal changes. All the animals were killed at 10 weeks of age to be considered as sexually mature. The population included 11 LTI and 10 STI Japanese quails obtained after 65 generations of Japanese quails which represents 33 years of breeding. The Japanese quails were euthanized with an intraperitoneal injection of pentobarbital (36 mg/100 g). After death, the quails were fixated using an intracardiac perfusion process with 400 mL of ice-cold 4% paraformaldehyde (PFA) solution in 0.1 M phosphate buffer for 10–20 min. Afterward, their heads were removed and preserved in PFA 4% at a temperature of 4 $^\circ$C for a duration of 8 weeks to continue the fixation process by immersion until scanning. The ex vivo brains were kept in their skulls to avoid any mechanical deformation during the fixation process, the skull being the most adequate container to reach this goal. Animal care and experimental treatments complied with the French Ministry of Agriculture guidelines for animal experimentation and European regulations on animal experimentation (86/609/EEC). They were performed in conjunction with the local animal regulation (authorized C37-175-1) of the French Ministry of Agriculture under the EEC directive and the ethics committee approval (Val de Loire, agreement Nř1789 and 1848).

The Japanese quails’ heads were plucked to avoid corruption of the MR images by susceptibility artifacts. The latter can be induced by the air bubbles trapped within the plumage and by the presence of oil in the plumage secreted by the uropygial gland (Jacob and Ziswiler 1982). The oil entails a fat peak on the NMR signal that needs to be canceled using a fat saturation preparation and thus increasing the scan duration. Two days before scanning, each sample was placed in 0.1 M phosphate-buffered saline (PBS) to achieve an optimal rehydration favorable to enhance the diffusion contrast (Thickman et al. 1983). At the time of scanning, each quail sample was put in a hermetically sealed Falcon^®^ tube containing Fluorinert^®^, an inert fluid that does not exhibit any NMR signal and conserves the hydration of the tissues. Finally, the Falcon^®^ tube was soundly fixed on an MRI bed originally built for rats during the scanning.

### MRI protocol

All MR acquisitions were performed on a preclinical Bruker BioSpec MRI scanner at 11.7 T. This imaging system is equipped with a powerful gradient set capable of delivering up to 780 mT/m with a slew rate of 9500 T/m/s allowing to reach very short echo times (less than 25 ms) to obtain images with a large signal-to-noise ratio, and offering the possibility of scanning the ex vivo samples with strong diffusion sensitizations (b-value of 4500 s/mm^2^ for the current scans). The imaging protocol included the acquisition of a high-resolution anatomical MRI dataset to create the cohort’s brain template, and the acquisition of a single-shell high angular resolution diffusion-weighted (HARDI) MRI dataset to infer the structural connectivity at the individual scale and then at the population scale. The volume of the Japanese quail’s brain is similar to that of the mice, thus advocating the use of a small bore preclinical ultra-high field (UHF) MRI system to scan the birds’ brains post-mortem. Acquisitions were performed using a Bruker ^1^H transmit–receive volume coil originally built for rats (40 mm inner diameter), where the full head of each quail could properly fit in. The temperature of the scanned sample was stabilized at room temperature (20 $^{\circ }$C) prior the scan. Then it was maintained at room temperature during the entire acquisition session using multiple pauses in the acquisition protocol that prevented the sample to heat up.

The anatomical MRI scans were performed using a conventional 2D T_2_-weighted spin-echo (SE) sequence with the following parameters: echo time TE = 16 ms, repetition time TR = 9 s, acquisition matrix = 160 $\times$ 250 $\times$ 170, spatial resolution = 150 $\times$ 150 $\times$ 150 $\upmu$m, 8 averages, 1 repetition, field of view = 24.0 $\times$ 37.5 $\times$ 25.5 mm, read bandwidth RBW = 65789.5 Hz, total scan duration of 2 h 45 min. The SE MRI dataset offered an enhanced contrast, facilitating the delineation of the deepest regions of interest (ROIs) by three independent neurobiologists.

The diffusion-weighted MRI scans were implemented using a 3D segmented echo planar imaging (EPI) pulsed gradient spin-echo (PGSE) pulse sequence. A single-shell high angular resolution diffusion imaging (HARDI) scheme was chosen to sample the diffusion Q-space along 75 diffusion-encoding directions uniformly distributed over the sphere at a b-value of 4500 s/mm^2^. Five T_2_-weighted volumes were also collected at *b* = 0 s/mm^2^ to normalize the diffusion-weighted signal and compute the corresponding diffusion decay. The other imaging parameters for this protocol corresponded to: echo time TE = 23.88 ms, repetition time TR = 250 ms, spatial resolution = 150 × 150 × 150 $\upmu$m, field of view = 30.0 × 40.0 × 26.0 mm, acquisition matrix = 150 × 200 × 130, read bandwidth RBW = 300 kHz, 18 EPI segments, pulse duration $\delta$ = 5 ms, diffusion time $\Delta$ = 12.3 ms, leading to a diffusion time of 10.6 $\upmu$s and to a total scan duration of 13 h 04 min.

### Image processing and analysis

The anatomical and diffusion-weighted datasets were analyzed using two pipelines. The first pipeline targets the creation of a reference anatomical template of the Japanese quail brain, and the second pipeline targets the construction of an atlas of anatomical connectivity. Since the creation of the anatomical template is not the main focus of the work presented in this paper, the anatomical pipeline will be described briefly, while the second pipeline which is the aim of this study is described in more detail below.

#### Anatomical template

**Fig. 1:**
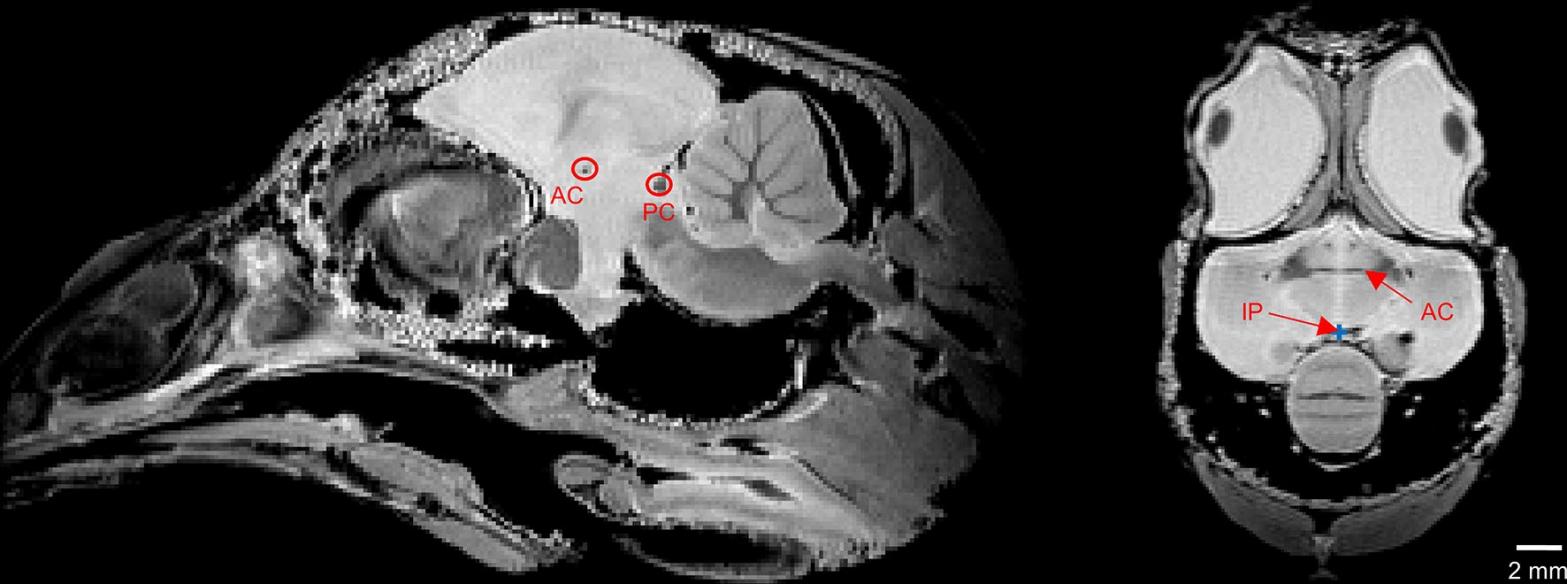
Anatomical images of subject 7133 LTI in the sagittal (on the left) and axial (on the right) views with an isotropic 150 $\upmu$m resolution showing three typical reference points used to define the AC–PC frame for each Japanese quail. These images are prior the reorientation in the AC-PC frame of the subject. *AC* anterior commissure, *PC* posterior commissure, *IP* interhemispheric point

To our knowledge, no MRI template has been published for the male Japanese quail. This situation advocated for the creation of a specific quail template. References in the literature (Güntürkün et al. 2013; Karten and Hodos 1967) suggest reorienting the bird’s head based on the midline of its beak. But this strategy does not really take into consideration the anatomy of the brain itself. An alternative strategy was proposed here to more specifically integrate the brain anatomy. To this aim, all the individual brains were manually segmented from their corresponding 150 $\upmu$m T_2_-weighted SE MRI scans. The extracted brains were then reoriented to align their anterior commissure (AC) and their posterior commissure (PC) in the same plane (see Fig. 1). It allowed the computation of a T_2_-weighted template using the antsMultivariateTemplateConstruction2.sh function from the Advanced Neuroimaging Tools (ANTs) toolbox (Avants et al. 2011).

**Fig. 2:**
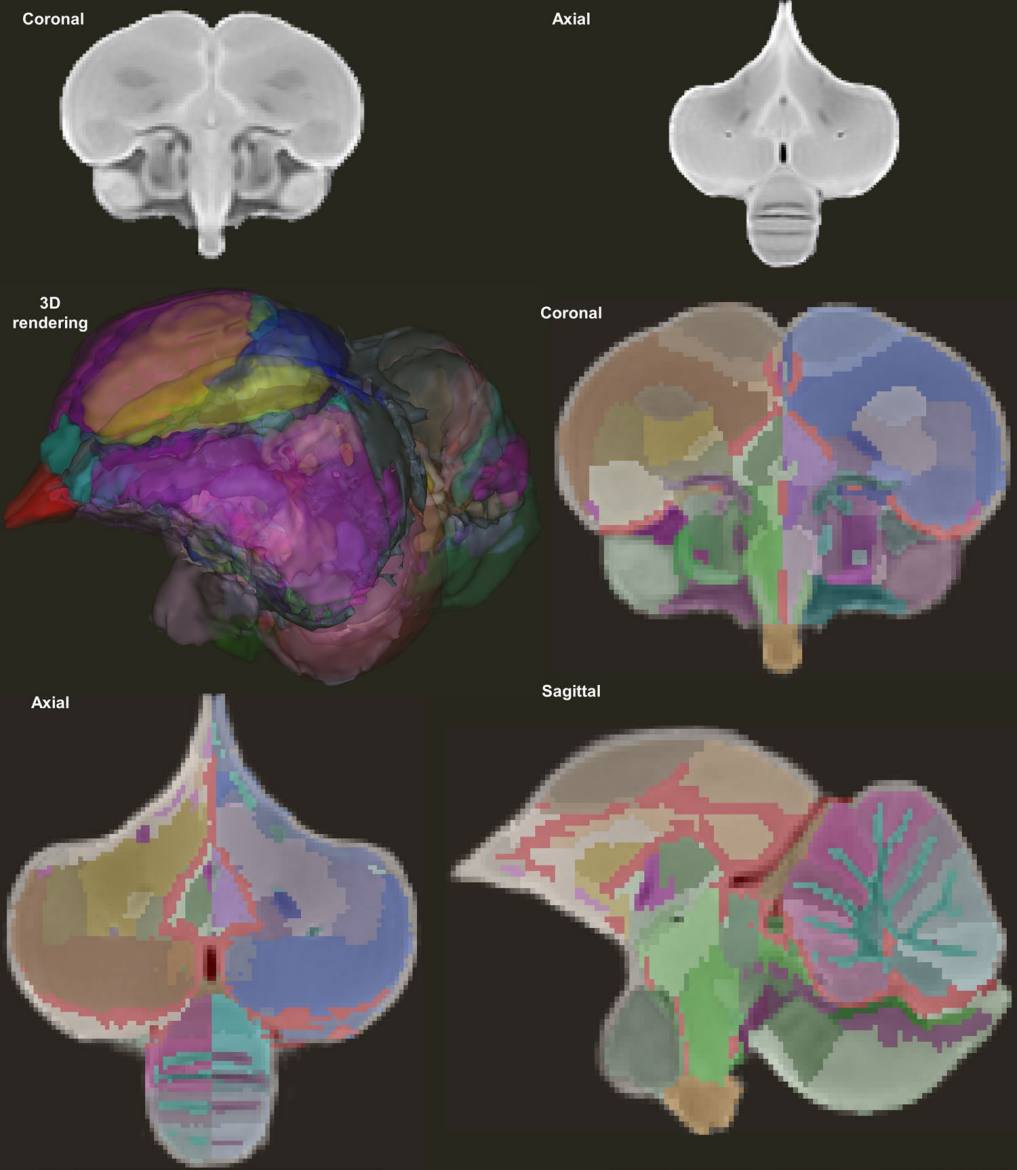
Template images obtained from the Japanese quail cohort. The template image in the coronal and axial views (at the top). The template image superimposed on the 195 delineated structures labeled and represented with distinct colors in the three spatial views as well as a 3D rendering of the template labels. These images are available at https://doi.org/10.5281/zenodo.4700523

The manual segmentation of the male Japanese quail’s anatomical substrates was realized by three independent neurobiologists on the template image, available on the online content entitled “Quail (*Coturnix japonica*) brain MRI template and whole-brain atlas” (https://doi.org/10.5281/zenodo.4700523). The structures were identified from the chick atlas (Kuenzel and Masson 1988; Puelles et al. 2018), but also based on similarities with other avians (pigeon, canary, starling and zebra finch) helping in the segmentation process (De Groof et al. 2016; Güntürkün et al. 2013, 2020; Poirier et al. 2008; Vellema et al. 2011). A specific pair of label and color was assigned to each structure, as shown in Fig. 2. As we will see next, the set of labeled structures facilitated the naming of the identified white matter bundles in the final structural connectivity atlas.

#### Diffusion MRI analysis

A specific analysis pipeline has been designed for the processing of diffusion-weighted MRI data, based on the use of the Ginkgo toolbox developed by the CEA/NeuroSpin team available at https://framagit.org/cpoupon/gkg. This pipeline integrates several consecutive stages of data processing from the individual scale to the group level. It includes the correction of imaging artifacts, the local modeling of the diffusion process, the inference of individual connectivity maps (or tractograms) using fiber tracking techniques, the registration of individual dMRI dataset to the Japanese quail template, and the segmentation of white matter bundles using advanced clustering approaches. These various steps are detailed below.

*Pre-processing*: dMRI datasets, typically acquired *in vivo* with single-shot EPI sequences, need to be corrected from all imaging artifacts such as susceptibility-induced geometrical and signal intensity distortions, eddy currents-induced geometrical distortions caused by the commutation of the strong diffusion gradients in single-shot EPI schemes, or even subject motion when they are acquired in vivo. These artifacts are mainly related to the long echo train used in single-shot EPI schemes making it extremely sensitive to any field inhomogeneity or motion. In our ex vivo case, dMRI was performed using an 18-segment multishot 3D EPI sequence on a UHF 11.7 T MRI system equipped with strong gradients (up to 780 mT/m at 9500 T/m/s). On the one hand, it allowed to benefit from a direct SNR increase due to the high static field (11.7 T). On the other hand, the fit allowed to benefit from a significant reduction of the echo time and echo train duration thanks to the combination of strong gradient magnitudes (780 mT/m) and a large number of segments (18 segments). Such an acquisition scheme led to the observation of almost no image distortion, thus limiting the pre-processing stage to the sole correction of the Rician noise corrupting the signal intensity. To this aim, a non-local means filter adapted to diffusion MRI was applied to the raw data as proposed in Buades et al. (2011). For each quail, a brain mask was manually delineated by three independent neurobiologists from the average 200 $\upmu$m T_2_-weighted MRI scan acquired at *b* = 0 s/mm^2^. Last, all the noise-free diffusion MRI scans were diffeomorphically co-registered to the template image using the ANTs toolbox (Avants et al. 2011). The process relies on the use of ODF images of all subjects to create an unbiased population template followed by a subject-to-template warps registration.

*Inference of the structural connectivity*: the inference of the structural connectivity of each individual relies on the combination of a local reconstruction method modeling the diffusion process and of a fiber reconstruction (or tractography) technique. Beyond the diffusion tensor (DTI, Basser et al. 1994) which is unable to model more than one population of fibers within a voxel, many high angular resolution diffusion imaging (HARDI) reconstruction techniques have been proposed in the literature to model the diffusion process locally. Such models are able to provide for each point of the brain the angular profile of the diffusion process (e.g., the orientation distribution function or ODF) or the angular profile of the fiber orientations (also called the fiber orientation distribution or FOD) from a set of diffusion-weighted measurements at the surface of a sphere (or shell) of radius equal to the diffusion sensitization *b*. It is out of the scope of this work to detail the plethora of existing reconstruction techniques, but the review article published by Tournier et al. (2011) gives a valuable overview of them. Among these models, reconstruction techniques involving deconvolution of the diffusion MRI signal with the impulse response of a homogeneous fiber population to the diffusion process are the most popular today (Tournier et al. 2007). In the case of this study, they were initially discarded because Japanese quails are deprived of a corpus callosum (Diekamp et al. 2005) which is representative of such a homogeneous fiber population, thus complicating the estimation of an adequate impulse response. It appeared more appropriate to use the analytical version of the Q-ball model (Descoteaux et al. 2007; Tuch 2004) which does not make any assumption about this impulse response, and which is based on a simple decomposition of the signal acquired on the sphere of radius *b* = 4500 s/mm^2^ onto a modified spherical harmonics basis with the robustness of the *L*-curve regularization factor to compute local ODFs. The small animal 11.7 T MRI system used to scan the quail brains is equipped with strong gradient coils that allow to obtain a b-value of 4500 s/mm^2^ with a very short echo time (below 24 ms) yielding sharp Q-ball ODFs without the need to apply any deconvolution.

The analytical Q-ball reconstruction was performed for each voxel of the brain mask using a spherical harmonics order of 8 with a regularization *L*-curve factor of 0.006, yielding for each individual an ODF map giving access to the local orientation of fibers. Inherited rotationally invariant Q-ball maps, such as the generalized fractional anisotropy (GFA) maps, were also computed, to further regularize fiber tracking techniques. A DTI reconstruction was also performed to compute other rotationally invariant maps including the fractional anisotropy (FA), apparent diffusion coefficient (ADC) and axial (AD) and radial diffusivities (RD).

Various fiber tracking (also called tractography) methods exist to infer the trajectories of axonal white matter fibers from dMRI-based ODF maps, including deterministic, probabilistic and global methods. It is also out of the scope of this study to discuss their commonalities and differences. The reader is invited to read the reference articles Neher et al. (2015) and Maier-Hein et al. (2017) for a general review of the different methods and tools available in the field. In this study, whole brain tractography was performed from the individual Q-ball ODF maps using the streamline regularized deterministic (SRD) technique proposed in Perrin et al. (2005) with the following parameters: creation of eight seeds per voxel of the brain mask, aperture angle of 30 $^\circ$, fiber length range of [0.1–100] mm, forward and backward integration step of 50 $\upmu$m, and anisotropy-based regularization factor of 0.12. All streamlines were stopped when they reached the boundaries of the previously mentioned brain mask, without any stopping criteria related to FA (or GFA) to avoid the definition of any empirical threshold. The fiber length range was tuned to discard fibers smaller than half the voxel size and to prevent the existence of infinite loops.

*Segmentation of white matter bundles*: one of the main purposes of this work is to establish an atlas of the Japanese quail structural connectivity. It consists of grouping the fibers obtained from diffusion-based tractography into bundles corresponding to the significant connection hubs of the Japanese quail’s brain. Fibre bundle segmentation methods can be grouped into two categories: supervised methods relying on a prior knowledge of the bundles, and unsupervised methods for which the bundles are not known a priori. The first category requires the definition of rules and pre-identified regions for each bundle that allow the selection of fibers when they pass through so-called intersection regions or the exclusion of fibers when they pass through so-called exclusion regions. Supervised methods were the first to be introduced, as they adopt an approach similar to that adopted by neuroanatomists during the last century to dissect long white mater bundles (Cajal 1911; Klingler 1935; Klingler and Gloor 1960). The latest refinements proposed to improve supervised methods consist in defining prior distributions of the neighboring anatomical structures of each pathway to further constrain the segmentation of white matter bundles such as TRACULA (TRActs Constrained by UnderLying Anatomy) (Yendiki et al. 2011).

However, it should be noticed that supervised approaches do not take into account the specific geometry of white matter bundles. A fiber bundle consists of a set of closely arranged parallel axonal fibers having a low to moderate curvature. This observation paves the way to the second class of unsupervised algorithms aiming at grouping fibers according to their distance (Corouge et al. 2004), which does not necessitate the definition of region-based anatomical priors and is suitable to investigate the structural connectivity of species without any knowledge of the existing fiber tracts. A good example of it is the human superficial connectivity that was poorly described by neuroanatomists, due to the difficulty its dissection represents. Defining a metric to measure the distance between fibers allows the application of clustering methods partitioning the fibers into clusters based on the computation of connectivity matrices that provide for any couple of fibers a measure of the affinity between them. Choosing the adequate distance is not trivial, as the quality of the resulting fiber clusters strongly depends on it.

Tractography methods can generate up to several millions of streamlines at the individual scale. Computing a connectivity matrix with such a sizable number of streamlines is not realistic from a computational standpoint. It is even worse when considering the plethora of streamlines available at the population level. To alleviate these computational bottlenecks, developing strategies to reduce the dimension of the problem becomes mandatory. An adequate dimension reduction strategy was proposed in Guevara et al. (2011) relying on two consecutive clustering steps: a first step at the subject level and a second step at the group level. Similar approaches were also devised in Garyfallidis et al. (2012) and Siless et al. (2018).

The structural connectivity of the Japanese quail has never been investigated broadly in the literature using diffusion MRI, even though some anatomical atlases published for the canary (Vellema et al. 2011) and the starling (De Groof et al. 2016) provide some insights about the expected connectivity of quails. This lack of preliminary data tipped the balance in favor of unsupervised techniques, and we propose here to extend the approach of Guevara et al. (2011) originally designed for humans to infer the structural connectivity of Japanese quails. As mentioned previously, the method relies on a threefold process: (1) a first clustering step operated at the individual scale to partition the fibers of a subject into small fascicles; (2) a second step performed at the population level to match inter-individual fascicles and create inter-individual clusters; (3) a third step to label inter-individual clusters and build the target structural connectivity atlas. It remains out of the scope of this study to detail the clustering approach proposed by Guevara et al. (2011). But for the sake of clarity, the three steps are summarized hereafter, highlighting the key parameters that need to be tuned accurately:

*Step 1: clustering of fibers at the individual scale*—white matter fibers were first separated according to the left hemisphere, right hemisphere and cerebellum, and then divided into eight groups corresponding to their length ranges from 0.64 to 49.06 mm, corresponding to the 2 and 98% boundaries of the cumulative fiber length histogram computed over the entire population. For each resulting fiber group, a density map was computed and thresholded to define a binary mask composed of the voxels crossed by at least five fibers. A *k*-means algorithm was then applied to subdivide the binary mask into random parcels of 27 voxels on average, contributing to the reduction of the problem dimension and enabling the computation of a connectivity matrix between the different parcels. Such a connectivity matrix was computed for each of the eight fiber length ranges, and for the left hemisphere, right hemisphere and cerebellum. The next step consisted in the application of a hierarchical clustering algorithm to the connectivity matrix to extract clusters of connected parcels. The obtained clusters were finally used to select the corresponding fibers from the original tractogram, assuming that a fiber belongs to a cluster if at least 31% of its trajectory intersects the cluster area. Clusters containing less than five fibers were discarded and every kept cluster was represented by a centroid defined as the fiber belonging to the cluster which depicts the smallest (symmetric mean of mean closest point) distance to the other fibers. In the end, a map of centroids was built that represents the set of fiber clusters segmented at the individual scale. This centroid map provides a sparse representation of the individual fiber cluster map and therefore further contributes to the reduction of the problem dimension. Table 1 summarizes the parameters applied for this individual clustering step.

**Table 1:** Set of intra-subject fiber clustering parameters used for the Japanese quail cohort

Parameter name	Value
Parcellation resolution	200 $\upmu$m
Average cluster size	6000 voxels
Minimum cluster size	300 voxels
Min. percentage of fiber length intersecting a cluster	31%
Length range of the fibers	0.64–49.06 mm
Number of length ranges	8
Minimum fiber count in a cluster	5

*Step 2: clustering of fibers at the population scale*—it consists in creating a second level of clusters of the centroids stemming from all the individuals. This sparse representation allows to keep the issue dimension reasonable, whatever the population size is. To this aim, the individual centroid maps were first co-registered to the template Japanese quail space, enabling the computation of an affinity matrix between centroids using the normalized pairwise distance integrating a correction to release the distance constraint when the minimum centroid length increases as proposed in Guevara et al. (2011) and adapted to the Japanese quail brain size. Centroid affinities were computed from the centroid distances using a Gaussian kernel yielding gathered into an affinity matrix. A hierarchical HDBSCAN clustering algorithm (Campello et al. 2013) was finally applied to the affinity matrix to extract centroid clusters at the group level (Table 2).

**Table 2:** Set of inter-subject fiber clustering parameters for the Japanese quail cohort

Parameter name	Value
Type of centroid distance	Normalized pairwise
Normalization factor nf	4.0 mm
Minimum centroid length $l_\mathrm{min}$	0.64 mm
Maximum centroid length $l_\mathrm{max}$	49.06 mm
Minimum normalized distance	0.01 mm
Maximum distance for the centroid affinity	2.0 mm
Maximum distance after exclusion	0.4 mm
Variance of the centroid affinity $\sigma ^{2}$	1600 mm$^2$
Centroid point count	21
Minimum population percentage	50%

*Step 3: bundle atlas construction*—a final stage performed in close collaboration with neuroanatomists involved regrouping inter-subject clusters to reconstruct white matter bundles plausible from an anatomical point of view. This last task was carried out semi-automatically. When the bundles were known to pass through regions that were well identified from the anatomical template of the Japanese quail, these regions were then used to select candidate centroids. Exclusion anatomical regions were also used to remove some of the candidates selected in the previous step that did not obviously belong to the targeted fiber bundle. At the end of this semi-automatic step, each bundle of interest was then made up of centroids representing fiber clusters stemming from all individuals and matched to the template space, which are then re-aggregated to form a new anatomical connectivity atlas of the male Japanese quail.

## Results

### Inference of the structural connectivity at the individual scale

#### Analytical DTI and Q-ball and DTI local modeling

**Fig. 3:**
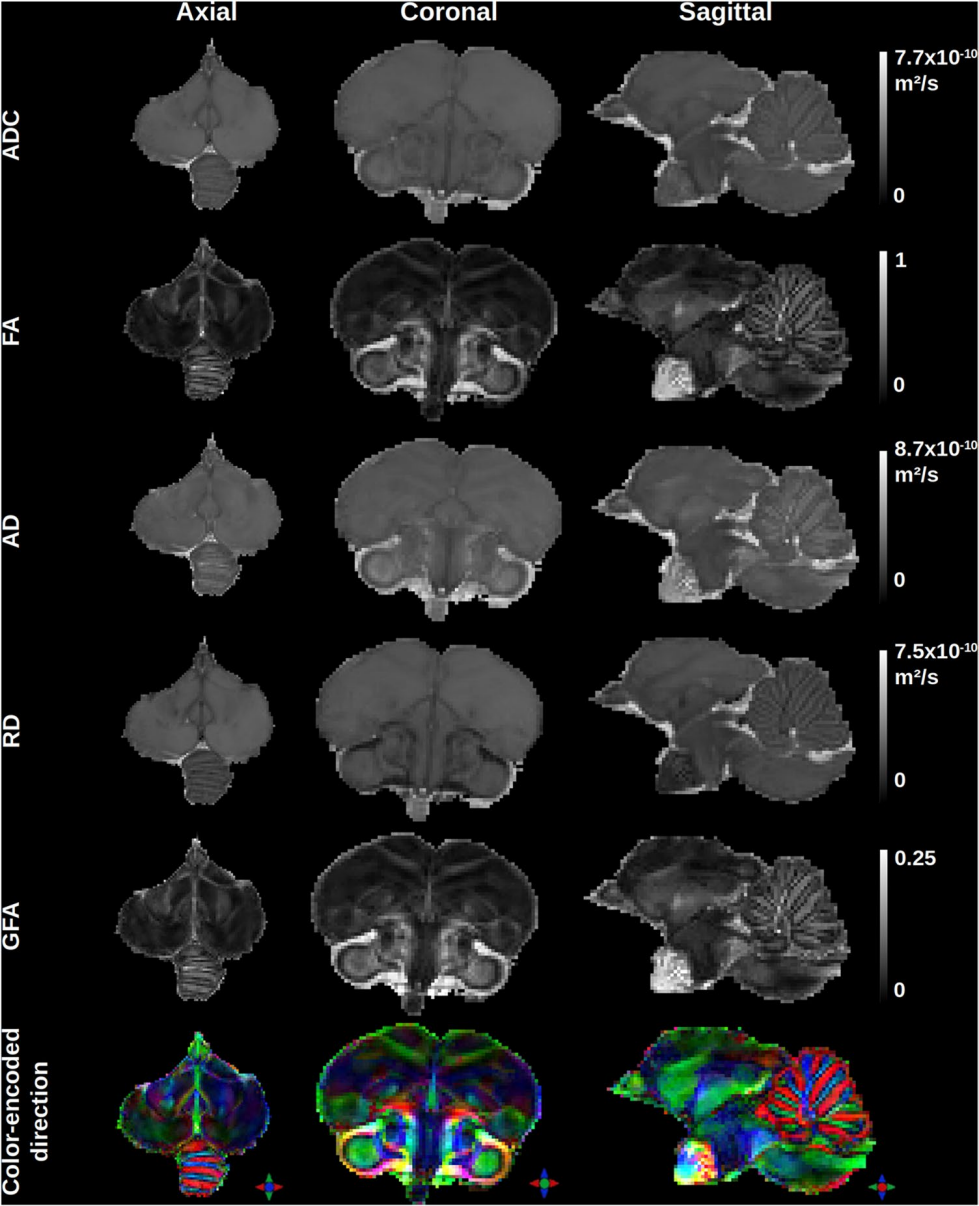
Representation of rotationally invariant DTI quantitative maps including (1st line) the apparent diffusion coefficient (ADC), (2nd line) the fractional anisotropy (FA), (3rd line) the axial diffusivity (AD), (4th line) the radial diffusivity (RD), and of analytical Q-ball quantitative maps including (5th lines) the generalized fractional anisotropy (GFA) and (6th line) the color-encoded direction (CED) map computed from a single-shell HARDI diffusion MRI acquisition performed on the brain of a Japanese quail at 11.7 T

The DTI and analytical Q-ball imaging (aQBI) local models of the diffusion process enabled obtaining quantitative maps that offer information on the microstructure of the quail’s brain as depicted in Fig. 3. Within the brain, the mean diffusivity (ADC) map depicts three modes corresponding to the gray matter (3.0 $\times$ 10$^{-9}$ mm$^2$/s), white matter (2.3 $\times$ 10$^{-9}$ mm$^2$/s) and CSF (6.5 $\times$ 10$^{-9}$ mm$^2$/s). The fractional anisotropy (FA) stemming from the DTI model ranges from 0 to 0.84, whereas the generalized fractional anisotropy (GFA) stemming from the Q-ball model ranges from 0.01 to 0.26. The color-encoded direction (CED) map also stemming from the Q-ball model reveals the main directions of the fiber populations and provides an overview of the homogeneous white matter bundles in the male Japanese quail brain.

#### Individual ODF maps and tractograms

**Fig. 4:**
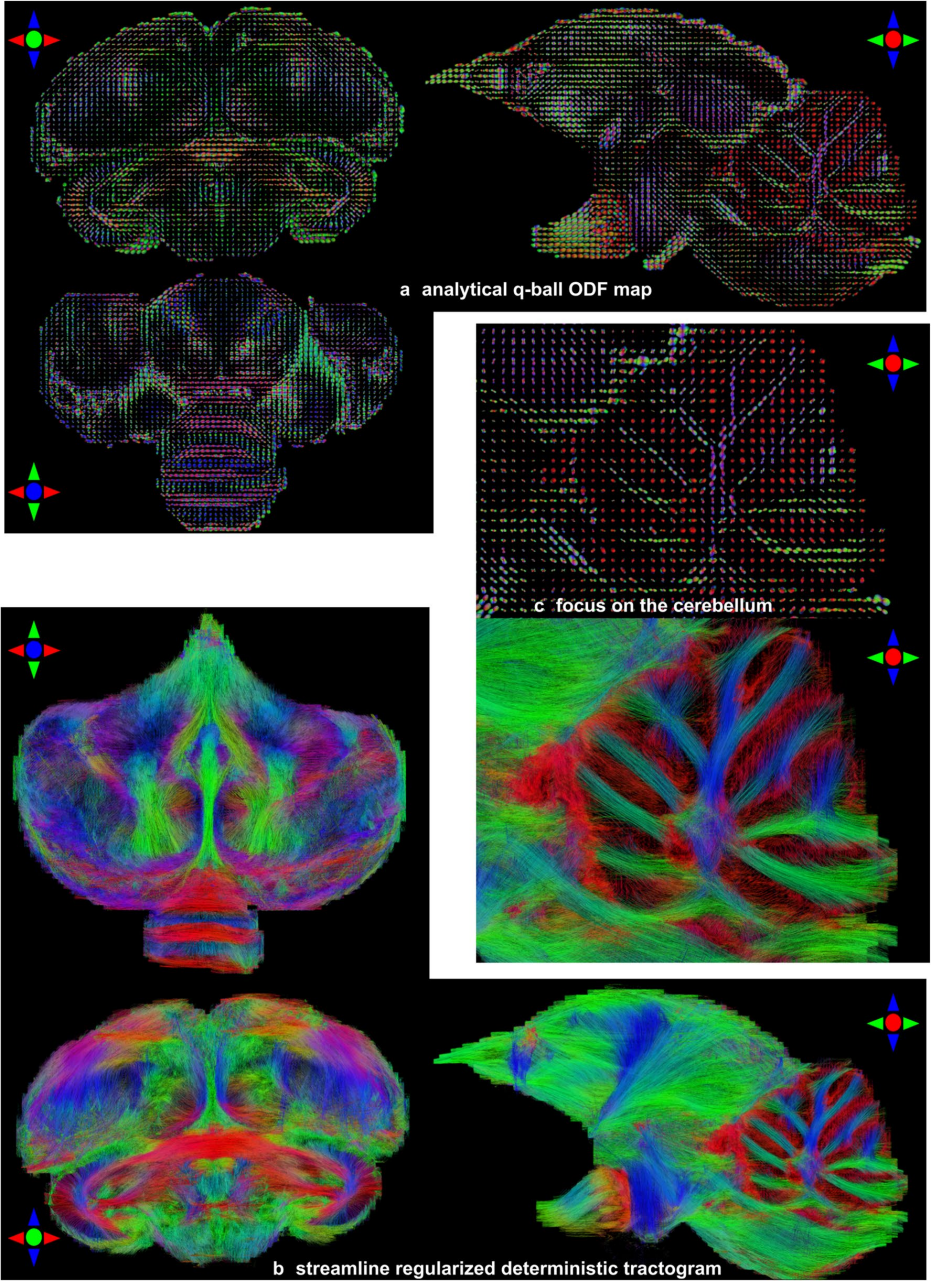
Orientation distribution function (ODF) maps of subject 7426 STI stemming from the analytical Q-ball model at spherical harmonics order 8 and corresponding tractogram obtained using an SRD tractography method. **a** Analytical Q-ball ODF maps. **b** Streamline regularized deterministic tractograms. **c** A zoom in ODFs and the tractogram located in the cerebellum

Figure 4 stresses the diffusion ODF map depicting various orientation profiles of the diffusion process, from configurations corresponding to the presence of a single fiber population represented by sharp single-lobe ODFs, to more complex configurations corresponding to the crossing of different fiber populations represented by multiple-lobe ODFs. A zoomed area over the cerebellum clearly shows its various lobules and the branching occurring at their basis, hence assessing the efficacy of the analytical Q-ball model to represent the underlying axon directions.

**Fig. 5:**
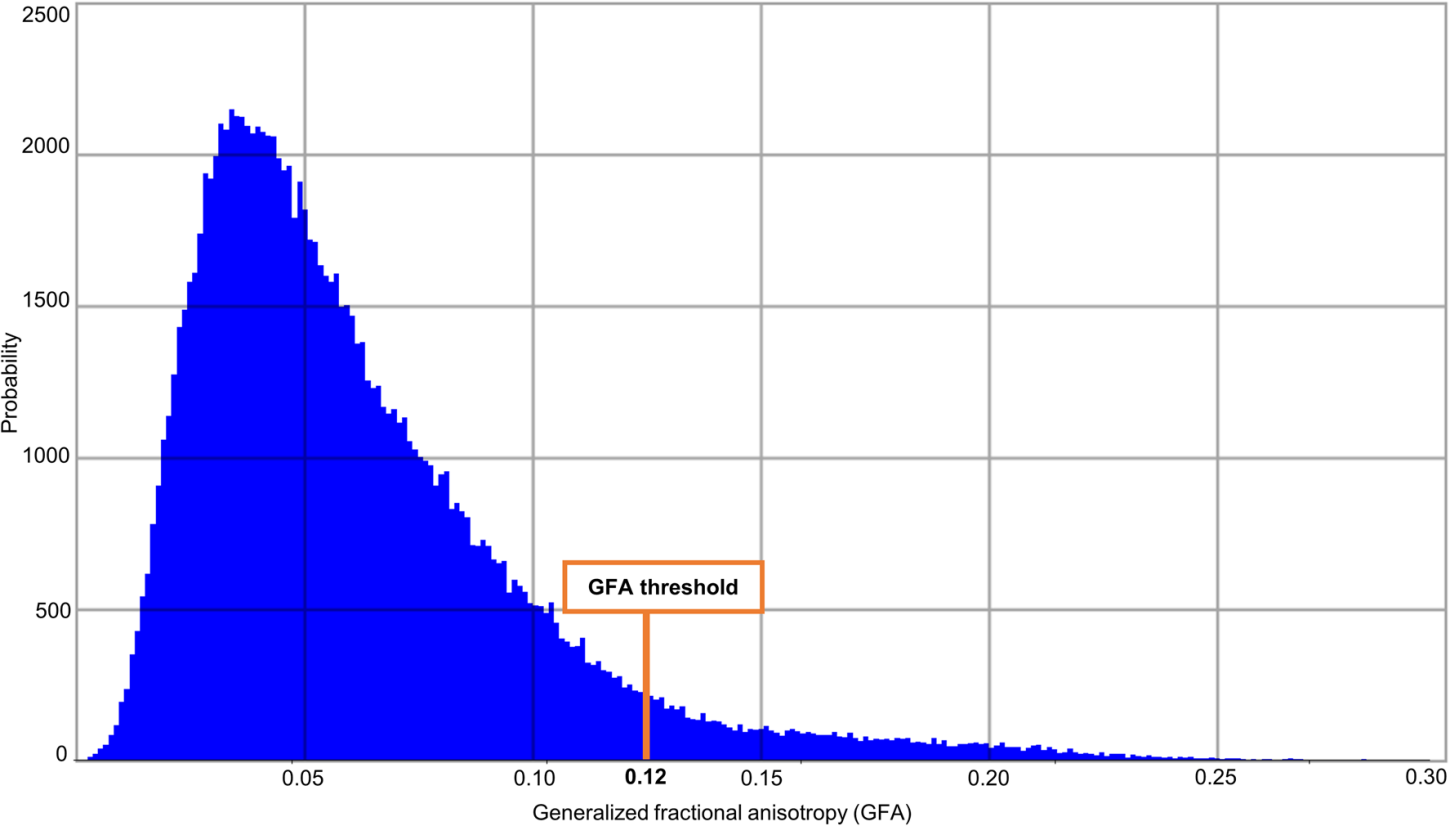
Histogram of generalized fractional anisotropy (GFA) for Japanese quail 7412 STI indicating the GFA threshold used for the entire cohort

To adjust the regularization factor of the tractography algorithms, an analysis of the GFA histogram provided in Fig. 5 is performed for each individual to compute a threshold value corresponding to 98% of its cumulative GFA histogram, yielding an average threshold of 0.12 across the population. This value was used to regularize the streamline construction as described in Perrin et al. (2005) and needs to be carefully chosen to avoid excessive regularization that would erase the actual curvature of streamlines.

Tractograms were then computed for each quail from the analytical Q-ball ODF maps using the SRD fiber tracking method, resulting in a large number of reconstructed streamlines per individual being 879036 on average with a standard deviation of 60039. The bottom part of Fig. 5 provides three axial, coronal and sagittal views of the reconstructed streamlines with a color-encoding system enhancing the representation of their local direction. Some large white matter bundles could qualitatively be identified and correspond to sets of tightly assembled streamlines creating connection hubs. For instance, the reconstructed streamlines within the cerebellar white matter clearly highlight the arbor vitae, called that way due to its branched, tree-like appearance. It was therefore natural to pursue the analysis of the quails’ individual tractograms using some automatic fiber clustering approaches.

### Fiber clustering on an individual scale

**Fig. 6:**
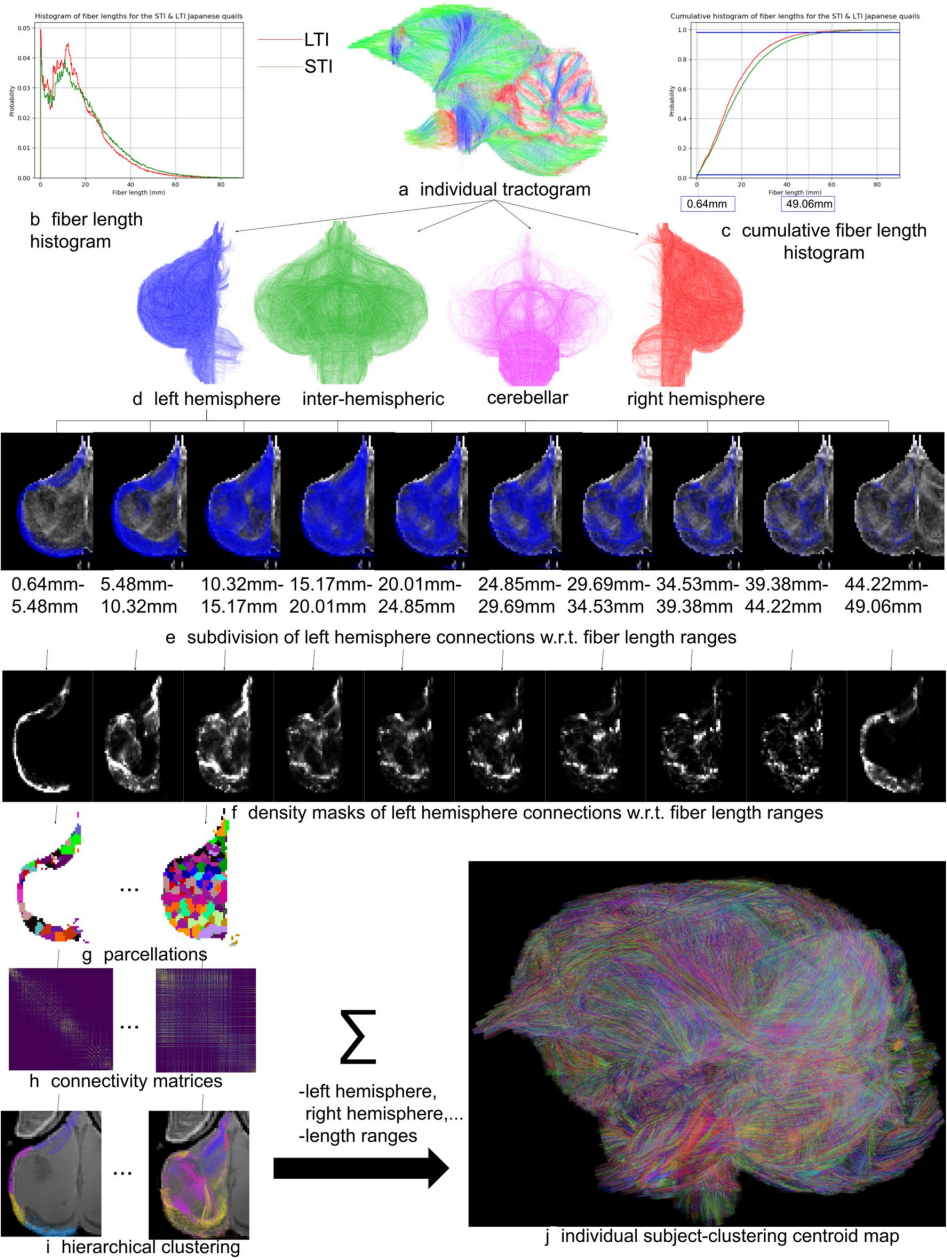
Fiber clustering at the individual scale relying on the processing of streamlines (**a**) corresponding to a fiber length range computed from fiber length histograms (**b**, **c**) and including: **d** the division of fibers according to their belonging to the left and right hemispheres, interhemispheric region or cerebellum, **e** the subdivision of each fiber set into subset of ten fiber ranges, **f** the computation of density masks corresponding to each subset, **g** the parcellation of the density mask, **h** the computation of connectivity matrices corresponding to each region and fiber length range, **i** the hierarchical clustering of fibers, and **j** the aggregation of results to individual cluster and centroid maps; each fiber cluster was attributed a specific color

A preliminary step before applying fiber clustering was to define an adequate fiber length range since clustering methods can be sensitive to spurious streamlines. To this aim, it appeared reasonable to discard very short streamlines typically corresponding to the result of aborted streamlining processes close or within the cortical ribbon and very long streamlines than are obviously not anatomically plausible. As depicted on Fig. 6b, c, histograms of the obtained fiber length across the 21 quails were computed, enabling the definition of two lower and upper thresholds corresponding to 2 and 98% of the cumulative histogram and yielding a 0.64–49.06 mm fiber length range to be considered.

Figure 6 also illustrates the intra-subject fiber clustering process for one of the quail brain. The subset of streamlines belonging to the 0.64–49.06 mm fiber length range was extracted (Fig. 6a) from the full tractogram and divided into four sets of streamlines respectively corresponding to the left hemisphere, the right hemisphere, the interhemispheric region and the cerebellum (Fig. 6d). Each of these subsets was then subdivided according to ten fiber length ranges (Fig. 6e) showing that the shortest streamlines remain superficial (probably belonging to short subcortical white matter bundles), whereas the longer ones mostly occupy the deep white matter area, with most of the intermediate to long white matter fibers belonging to the 10.32–34.53 mm range. For each fiber length range, a fiber density mask was computed as depicted in Fig. 6f, then thresholded and followed by the computation of a parcellation of the resulting binary mask using a *k*-means algorithm to create parcels of 27 voxels on average (Fig. 6g). A connectivity matrix was then computed from each parcellation and subset of streamlines (Fig. 6h), followed by the application of a hierarchical clustering algorithm to create fiber clusters for the current fiber length range (Fig. 6i). Finally, the fiber cluster sets obtained for the different length ranges and hemispheres are aggregated to create the target individual final cluster map (Fig. 6j), plus a centroid map that corresponds to a light representation of it required for the next stage.

As shown in Fig. 6, each individual cluster map provided a set of fascicles, each of which was populated by a reduced set of fibers sharing similar trajectories and being close to each other. All the fibers belonging to a cluster were represented using a unique color assigned to the whole fascicle, with distinct colors assigned to the different clusters. A visual comparison revealed the existence of similar fascicles between two individuals, and also highlighted the possibility to establish larger white matter fiber bundles coherent between subjects by aggregating subsets of clusters for an individual, noting that the number of clusters to be aggregated may differ from one individual to another. Regardless of its belonging line, this first clustering step provided 79207 fiber clusters on average with a standard deviation of 26713 fiber clusters between individuals for the applied SRD tractography approach.

### Fiber clustering at group level

**Fig. 7:**
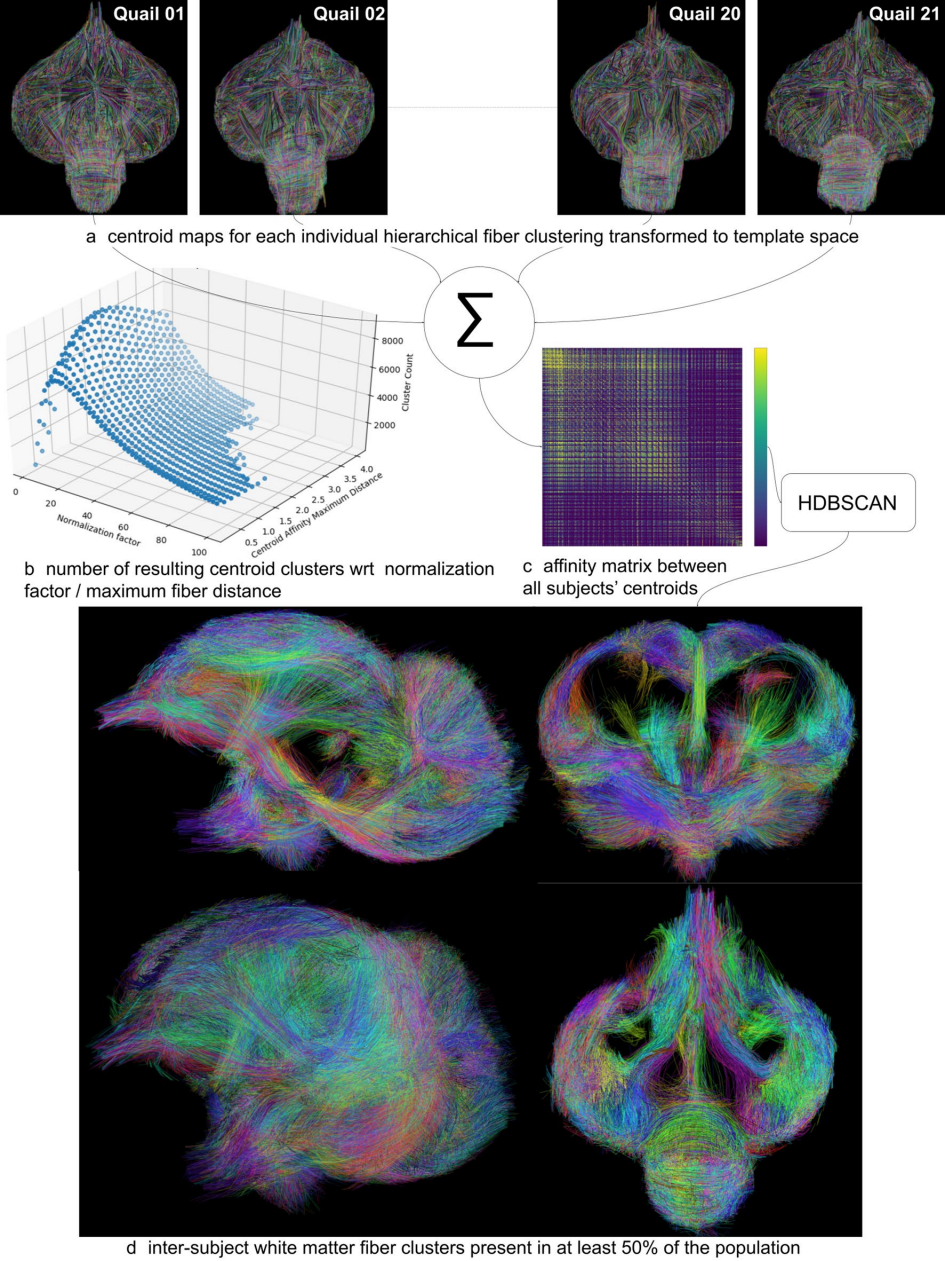
Fiber clustering at the population level relying on four consecutive steps including: **a** the diffeomorphic transformation of the individual centroid maps to the template Japanese quail space, **b** the computation of the optimum distance correction parameters (nf, CAMD) = (4.0 mm, 2.0 mm) using a grid-based optimization of the convex landscape corresponding to the number of created clusters, **c** the computation of the affinity matrix from centroids stemming from all the individuals, **d** the hierarchical HDBSCAN clustering of the affinity matrix providing inter-subject centroid clusters found in at least $50\%$ of the population; each centroid cluster was attributed a specific color

All the centroid maps obtained from the fiber clustering step performed at the individual scale were registered to the template space using the non-linear transformation computed from the ANTs toolbox (Fig. 7a), and were aggregated in a population-wise centroid map, before proceeding to the fiber clustering step at the group level.

The normalized pairwise distance proposed in Guevara et al. (2011) implements a correction of the distance between two centroids with respect to their minimum length. This correction relies on the tuning of two parameters corresponding to a normalization factor *nf* and a maximum distance between centroids *CAMD*. The number of inter-subject fiber clusters strongly depends on those two distance parameters. A preliminary study was conducted using a subset of four individuals to establish the optimum parameters, including two STI quails and two LTI quails. This study consisted in the evaluation of the obtained cluster number for a discrete $10 \times 10$ grid of (*nf*, *CAMD*) values covering two $[1.0\,mm; 100.0\,mm]$ and $[0.1\,mm; 4.0\,mm]$ ranges of values for nf and CAMD, respectively. Figure 7b shows the corresponding 3D surface plot for the SRD approach, depicting a convex landscape of the cluster count with respect to the nf and CAMD parameters. It allowed to get the optimal settings (nf, CAMD) = (4.0 mm, 2.0 mm).

Using this optimized tuning, the affinity matrix was computed as shown in Fig. 7c, providing the set of centroid clusters depicted in Fig. 7d that represents white matter fascicles present in at least $50\%$ of the cohort (Table 3).

**Table 3:** The number of clusters generated by the inter-subject fiber clustering steps from the SRD tractography method for the entire cohort depending on the located region of the brain

Brain location	Number of clusters (SRD)
Left hemisphere	7247
Right hemisphere	7206
Cerebellum	4321
Interhemispheric region	3105

*SRD* streamline regularized deterministic

### Atlasing

**Fig. 8:**
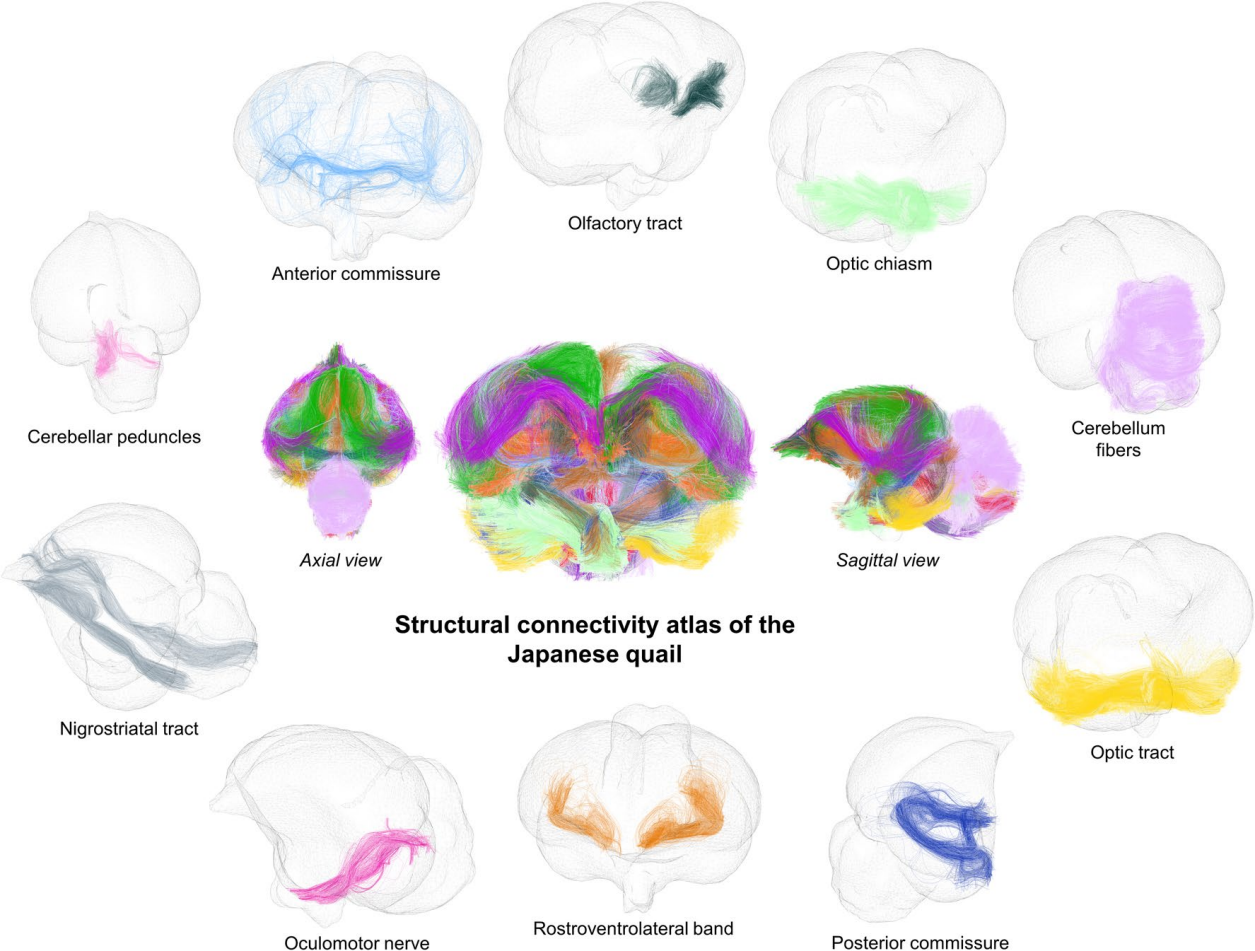
The structural connectivity atlas of the Japanese quail based on streamline regularized deterministic tractography and represented on a 3D mesh image of the template. The 34 identified white matter bundles are shown here in different colors as fibers of the clusters they belong to. The first set of white matter bundles shown here includes the olfactory tract, optic chiasm, cerebellum fibers, optic tract, posterior commissure, rostroventrolateral band, oculomotor nerve, nigrostriatal tract, cerebellar peduncles and anterior commissure

**Fig. 9:**
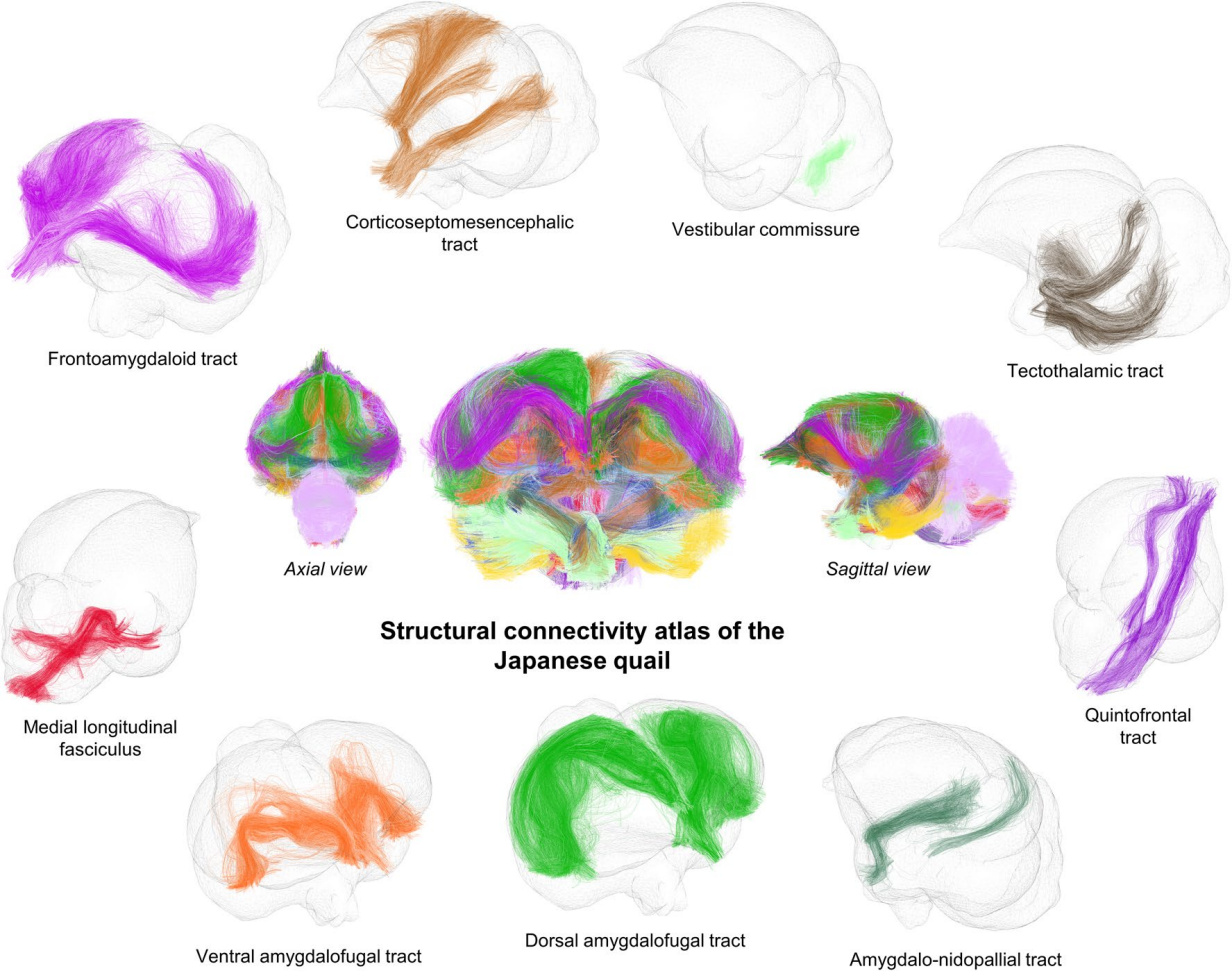
The structural connectivity atlas of the Japanese quail. The second set of white matter bundles shown here includes the vestibular commissure, tectothalamic tract, quintofrontal tract, amygdalo-nidopallial tract, dorsal amygdalofugal tract, ventral amygdalofugal tract, medial longitudinal fasciculus, frontoamygdaloid tract and corticoseptomesencephalic tract

This last semi-manual step consists in a consolidation of these individual clusters to point out the anatomically relevant white matter bundles. The final structural connectivity atlas shown in Figs. 8 and 9 illustrates 34 major fiber tracts in which labeling was realized in collaboration with neurobiologists to ensure a coherence with the 195 neuroanatomical structures identified from the template previously presented. Some of the identified structures were used to both select and group inter-subject fiber clusters and to name the 34 resulting white matter bundles listed below. We can partition the fiber tracts into three groups alike humans: commissural, projection and association fiber tracts.

The commissural fiber tracts refer to the anterior commissure [the only direct intertelencephalic pathway (Ehrlich and Mills 1985)], the posterior commissure (serves auxiliary visual functions (Stanic et al. 2010), the vestibular commissure [part of the flight control system (Bilo and Bilo 1978)], and the optic chiasm (initiates visual pathways).

The projection fiber tracts gather the corticoseptomesencephalic tract [descending pathway that innervates thalamic and brainstem nuclei (Güntürkün et al. 2013)], the medial longitudinal fasciculus (part of the motor system), the nigrostriatal tract [major dopaminergic pathway involved in expectation and delivery of food rewards (Ogura et al. 2015)], and the quintofrontal tract [afferent component in the control of mandibulation (Kuenzel 1982)].

The association fiber tracts correspond to the cerebellar peduncles (part of the motor system), the cerebellum fibers (part of the motor system), the frontoamygdaloid tract (part of the reward circuit (Kalenscher et al. 2005)), the olfactory tract (part of the olfactory system), the optic tract (part of the visual system), the oculomotor nerve (part of the visual system), the rostroventrolateral band (part of the visual system), the tectothalamic tract (part of the tectofugal system and thus motion detection (Wu et al. 2000)), the amygdalo-nidopallial tract which connects the amygdaloid taenial nucleus, amygdala, parataenial area, nidopallium and arcopallium amygdaloid complex (AAC), the ventral amygdalofugal tract which connects the AAC, bed nucleus of stria terminalis, striatum, nidopallium, mesopallium and ventral hyperpallium, the left-hemisphere (lh) dorsal amygdalofugal tract which connects the AAC, nidopallium and apical hyperpallium (only existing in the left hemisphere), and the right-hemisphere (rh) dorsal amygdalofugal tract which connects the AAC, mesopallium, nidopallium and ventral hyperpallium (only existing in the right hemisphere).

Table 4 distinguishes the structural connectivity atlas fiber tracts according to the part of the brain they belong to. Among the 34 white matter bundles of the structural connectivity atlas, 28 of them have their names based on the latest version of the chick atlas (Puelles et al. 2018). There are six fiber tracts, namely the amygdalo-nidopallial tracts, the ventral amygdalofugal tracts, and the lh and rh dorsal amygdalofugal tracts, which names were based on the main neuroanatomical structures they connect as they were not clearly identified in the avian nomenclature. Despite their sightly different pathways, we chose to assign the same name for lh and rh dorsal amygdalofugal tracts, considering that the avian brain lateralization (namely left/right asymmetry) may be the reason for this disparity.

**Table 4:**
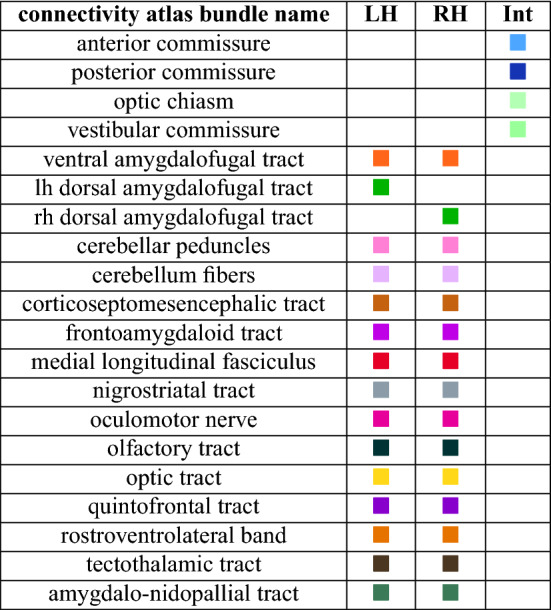
Table sorting the structural connectivity atlas fiber bundles according to the part of the brain they belong to

The colored squares have the same color as the corresponding bundles in the presented connectivity atlas of the Japanese quail in Figs. 8 and 9

*Int* interhemispheric region, *LH* left hemisphere, *RH* right hemisphere

## Discussion

### Connectivity-based comparisons between the two lines

No variance in the brain volume among the cohort was observed, with similar volumes found for the STI and the LTI quails. Therefore, analyses of variance were conducted on the 21 intra-subject fiber clusterings, based on the SRD tractograms at the root of the connectivity atlas and by applying *T* tests (*p* value < 0.05). We observed a line effect in favor of the STI line with a higher fiber average density than the LTI line. There were slightly more fibers in the left hemisphere considering the whole cohort as well as each line. This left/right effect was even more accentuated for the STI quails than the LTI ones. The cerebellums of the LTI quails appeared to have a greater fiber density than the STI quails, while the two lines were equivalent regarding the interhemispheric fibers. Therefore, the LTI line has a less dense connectivity in both hemispheres than the STI line. This discrepancy could reflect poorer connections related to the emotivity of the LTI line. Further investigations regarding the white matter bundles involved in the emotionality trait of the Japanese quail could validate the causes of this connectivity-based difference between the STI and LTI quails of this study.

### The brain lateralization of the Japanese quail

The functional brain lateralization is a common trait for all birds (Jonckers et al. 2015; Rogers 2008). The final structural connectivity atlas presented in Figs. 8 and 9 seems to mirror the brain lateralization of the Japanese quail on a structural connectivity level. We indeed counted 708 fiber clusters in the left hemisphere, 577 fiber clusters in the right hemisphere, 521 fiber clusters in the cerebellum and 145 interhemispheric fiber clusters. It has been defined that there is a domestic chick (*Gallus domesticus*) brain lateralization caused by the exposure to natural light in ovo (Wichman et al. 2009). Our findings suggest the brain lateralization may persist even for the studied Japanese quails that come from years of dark-incubated breeding. They are in conjunction with Costalunga et al. (2021) study that addresses the presence of multiple light-independent lateralization effects in the visual Wulst of the domestic chick, based on neural responses. These light-independent lateralization effects could then lead to an asymmetrical distribution of fibers in the Japanese quail’s brain.

Furthermore, this tendency reappears on a volumetric level. The chick’s left eye is specialized in responding to small changes in any of a variety of stimulus properties (Vallortigara and Andrew 1991) and is preferentially used in dangerous situations (Rogers 1995). As mentioned in Richard (2000), a higher volume of the right-hemisphere arcopallium amygdaloid complex (AAC) was noticed in this study, plus a larger number of fibers crossing the right optic tract and the right-hemisphere AAC compared to their left counterparts. From there, observations were conducted on the potential motor fiber connections triggered in those dangerous situations. However, no fiber connection in the structural connectivity atlas and on the individual scale was found between the AAC and the periaqueductal gray (PAG), the mesencephalic reticular formation (MRF) or the lateral pontine nucleus (LPN), as hypothesized in Saint-Dizier (2008), which could have reflected the motor pathway controlling fear-related behaviors of the Japanese quail. The 200 $\upmu$m isotropic resolution of the dMRI dataset acquired at 11.7 T may have not been enough to confirm the existence of such fiber connections. Only volumetric differences stood out, according to the performed t-tests (p-value < 0.05), where the MRF and PAG (dorsolateral and medio ventral parts) are significantly bigger for the STI quails than the LTI quails. Considering the entire cohort, the left-hemisphere LPN and medio-ventral parts of the PAG are wider than on the right-hemisphere side. The opposite effect is observed for the MRF and the dorsolateral parts of the PAG. Consequently, a direct link on the one hand between connections of the right optic tract and AAC, and on the other hand between the aforementioned anatomical structures and their brain volumes must be confirmed with further functional and neurochemical studies.

### Comparison to existing studies on bird structural connectivity

The presented Japanese quail’s structural connectivity MRI-based atlas was compared to that of the canary (Vellema et al. 2011), the starling (De Groof et al. 2016) and the pigeon (Güntürkün et al. 2013) publications. The Japanese quail’s atlas exhibits the highest number of labeled fiber tracts compared to the canary, the starling or pigeon atlases that, respectively, present 10, 12 and 8 fiber tracts. We may observe similarities between the Japanese quail and the canary, as well as the Japanese quail and the starling anatomical connectivity atlases. These commonalities, based on the nomenclature of the fiber tracts, are listed in Table 5.

**Table 5:** Table comparing the bundle names of the present structural connectivity atlas to those from the canary (Vellema et al. 2011) and the starling (De Groof et al. 2016)

Japanese quail bundle name	Canary bundle name	Starling bundle name
Anterior commissure	Anterior commisura (CoA)	Anterior commissure (CoA)
Posterior commissure	Posterior commisura (CoP)	Posterior commissure (CoP)
Optic chiasm	X	X
Vestibular commissure	X	X
Ventral amygdalofugal tract	X	X
lh dorsal amygdalofugal tract	X	X
rh dorsal amygdalofugal tract	X	X
Cerebellar peduncles	X	X
Cerebellum fibers	X	X
Corticoseptomesencephalic tract	Septopalliomesencephalic tract (TSM)	Septopalliomesencephalic tract (TSM)
Frontoamygdaloid tract	Frontoarcopallial tract (FA)	Frontoarcopallial tract (FA)
Medial longitudinal fasciculus	X	X
Nigrostriatal tract	X	X
Oculomotor nerve	Oculomotor nerve (3N)	Oculomotor nerve (3N)
Olfactory tract	X	X
Optic tract	Optic tract (OT)	Optic tract (Opt)
Quintofrontal tract	Quintofrontal tract (QF)	Quintofrontal tract (QF)
Rostroventrolateral band	X	X
Tectothalamic tract	X	X
Amygdalo-nidopallial tract	X	X

*X* no matching correspondence with the Japanese quail fiber tracts

Table 5 highlights the fact that the majority of fiber tracts regarding the canary and the starling were also found for the Japanese quail. Only the medial forebrain bundle (MFB), the occipito-mesencephalic tract (OM) and the lateral forebrain bundle (LFB) of the canary and the starling were not found and labeled for the Japanese quail. The names provided for the canary fiber tracts in Vellema et al. (2011) and for the starling in De Groof et al. (2016) are more specific to those detailed in Güntürkün et al. (2013) for the pigeon, making the comparison easier with the Japanese quail. Nonetheless, the major groups of pathways described in Güntürkün et al. (2013) (visual, somatosensory, auditory pathways and descending projections) were also found for the Japanese quail. For instance, we observed tectofugal and thalomofugal pathways for the Japanese quail such as the tectothalamic tract which exhibits projections from the tectum optic that terminate in the thalamic nuclei. The latter correspond to the prethalamic-thalamic area, dorsolateral anterior nucleus of the thalamus, dorsomedial anterior thalamic nucleus and dorsal paraventricular nucleus of the thalamus according to the presented anatomical atlas of the male Japanese quail available on Zenodo (https://doi.org/10.5281/zenodo.4700523).

This concordance between the anatomical connectivity of these three birds suggests a validity of the presented fiber tracts for the male Japanese quail. Nevertheless, there are some white matter bundles that were identified for the canary, the starling and the pigeon that were not found for the Japanese quail, which suggests that these fiber tracts may be harder to detect from the acquired T_2_-weighted scans. Likewise, a further histological validation remains necessary to soundly validate the trajectories of the male Japanese quail white matter bundles, especially those that were only found for the Japanese quail.

### Future directions

The designed male Japanese quail’s structural connectivity atlas ushers the deep investigation of its various functional circuits, for instance those involved in the emotionality trait of that species. The established post-mortem imaging protocol assesses the added value of preclinical MRI systems benefiting from both ultra-high magnetic fields and powerful gradients to map the anatomical substrates of avian brains. The proposed post-processing pipeline also showcases its robustness to map ex vivo the anatomical circuits of the Japanese quail. The imaging protocol and the post-processing pipeline can still be enhanced. The use of stronger magnetic fields (such as 15.4 or 17.2 T) and higher gradient magnitudes (1000 mT/m) could improve the spatio-angular resolution of individual anatomical and diffusion MRI dataset. For instance, it would allow to reach a mesoscopic resolution of 100 $\upmu$m to explore finer anatomical structures. It would also enable the use of ultra-high diffusion sensitizations (*b* > 10,000 s/mm$^2$) to facilitate the imaging of finer fascicles that are not accessible with the current protocol, such as the connections between the AAC and the PAG, the MRF and the LPN, as hypothesized by Saint-Dizier (2008).

Methodological improvements are also possible at the level of the local model and the fiber tracking technique used. The choices made for this study were motivated by the desire to use relatively simple and straightforward approaches in the first instance. The knowledge acquired during this study of the anatomical connectivity of the Japanese quail would now enable the use of more sophisticated local reconstruction techniques such as the MSMT-CSD (multi-shell multi-tissue constrained spherical deconvolution) model (Jeurissen et al. 2014), since strong white matter bundles could be used to infer the impulse response of fibers to the diffusion process. However, this approach would require a modification of the imaging protocol to sample the diffusion-weighted signal over several spheres of Q-space. Such a multiple shell acquisition scheme would allow further characterizations of the microstructure of the white matter fiber bundles present in the atlas by allowing, for example, the quantification of their axonal density or the orientation dispersion of their axons using the Bingham-NODDI model (Tariq et al. 2016). Furthermore, the use of probabilistic or global fiber tracking techniques would also contribute to help in the reconstruction of finer fiber bundles such as the aforementioned connections between the AAC and the PAG, the MRF and the LPN.

The present study proposes a new atlas of the anatomical connectivity of the male Japanese quail with reconstructed clusters present in at least 50% of the cohort, which constitutes a first validation step. However, further validation steps are still necessary, requiring the use of microscopic acquisitions on histological sections from a few individuals to confirm the mappings resulting from the analysis of diffusion-weighted MRI data. Among these methods, polarized light imaging (PLI, Axer et al. 2011), based on the exploitation of the birefringence of the myelin sheaths surrounding the axons, is particularly well suited to carry out this type of validation, as illustrated by the recent work carried out on the pigeon (Stacho et al. 2020) to establish a cortex-like canonical circuit in the avian forebrain.

Last, the designed structural connectivity atlas only used male subjects. It would be valuable to scan the brains of adult female Japanese quails with this imaging protocol so as to enrich the anatomical connectivity established here and to have a global understanding of the connectivity of the entire species. Moreover, it could be interesting to evaluate the feasibility of scanning Japanese quails in vivo at 11.7 T to generate functional MRI data, using a suitable functional paradigm involving the fear-related circuit.

## Conclusion

This study provides the first structural connectivity atlas of the male Japanese quail (*Coturnix japonica*) established using ultra-high field diffusion MRI at 11.7 T with advanced tractography and fiber clustering methods. It is composed of 34 white matter bundles reproducible between individuals, that correspond to connection hubs. Similar to what has already been done for the human brain, this novel atlas allows to explore the diversity of the structural connectivity of the male Japanese quail. Moreover, it enables to automatically segment the white matter bundles of any new quail individual, in order to better explore the functional networks of the male Japanese quail. This study notably focused on the structural connectivities of two lines of Japanese quails, showing significant differences between the short tonic immobility (STI) and the long tonic immobility (LTI) lines, in terms of morphometry of their neuroanatomical structures, and connectivity between the involved structures. This work paves the way to comprehend how the anatomical structures of the Japanese quail communicate and it provides an important tool for future animal structural connectivity studies.

## Data Availability

The template image, the anatomical atlas and its corresponding labeled structures are freely available on Zenodo (10.5281/zenodo.4700523). The final anatomical connectivity atlas that supports the findings is freely available on Framagit (https://framagit.org/cpoupon/gkg/-/tree/master/dmri/share/data/dmri/species/Quail).

## Declarations

### Code availability

The entire pipeline used to design the structural connectivity atlas is freely available on Framagit (https://framagit.org/cpoupon/gkg).
